# Desiccation resistance in tropical insects: causes and mechanisms underlying variability in a Panama ant community

**DOI:** 10.1002/ece3.2355

**Published:** 2016-08-08

**Authors:** Jelena Bujan, Stephen P. Yanoviak, Michael Kaspari

**Affiliations:** ^1^ Department of BiologyGraduate Program in Ecology and Evolutionary BiologyUniversity of OklahomaNormanOklahoma; ^2^ Department of BiologyUniversity of LouisvilleLouisvilleKentucky; ^3^ Smithsonian Tropical Research InstituteBalboaRepublic of Panama

**Keywords:** Body size, canopy, CT_max_, thermal tolerance, VPD, water content, water loss

## Abstract

Desiccation resistance, the ability of an organism to reduce water loss, is an essential trait in arid habitats. Drought frequency in tropical regions is predicted to increase with climate change, and small ectotherms are often under a strong desiccation risk. We tested hypotheses regarding the underexplored desiccation potential of tropical insects. We measured desiccation resistance in 82 ant species from a Panama rainforest by recording the time ants can survive desiccation stress. Species' desiccation resistance ranged from 0.7 h to 97.9 h. We tested the desiccation adaptation hypothesis, which predicts higher desiccation resistance in habitats with higher vapor pressure deficit (VPD) – the drying power of the air. In a Panama rainforest, canopy microclimates averaged a VPD of 0.43 kPa, compared to a VPD of 0.05 kPa in the understory. Canopy ants averaged desiccation resistances 2.8 times higher than the understory ants. We tested a number of mechanisms to account for desiccation resistance. Smaller insects should desiccate faster given their higher surface area to volume ratio. Desiccation resistance increased with ant mass, and canopy ants averaged 16% heavier than the understory ants. A second way to increase desiccation resistance is to carry more water. Water content was on average 2.5% higher in canopy ants, but total water content was not a good predictor of ant desiccation resistance or critical thermal maximum (CT_max_), a measure of an ant's thermal tolerance. In canopy ants, desiccation resistance and CT_max_ were inversely related, suggesting a tradeoff, while the two were positively correlated in understory ants. This is the first community level test of desiccation adaptation hypothesis in tropical insects. Tropical forests do contain desiccation‐resistant species, and while we cannot predict those simply based on their body size, high levels of desiccation resistance are always associated with the tropical canopy.

## Introduction

Small ectotherms are often at risk of desiccation given their high surface area to volume ratio, proportionately low fat storage, and relatively high metabolic rate (Gibbs 2003; Harrison et al. 2012). Desiccation resistance – the ability for an organism to reduce water loss – is thus a useful trait in small ectotherms, especially in light of predicted increases in the frequency and severity of droughts (IPCC 2014). Tropical forests contain a large fraction of Earth's species, but, perhaps due to their high relative humidity, little attention has been given to the patterns of desiccation resistance in tropical arthropods (Stanley and Parsons 1981; Karan et al. 1998; Hoffmann et al. 2003; Lapinski and Tschapka 2014). Here, we examine the patterns and mechanisms of desiccation resistance among 82 species of tropical ants from a diverse Panama rainforest community.

The most basic hypothesis for the distribution of desiccation resistance, which we call the desiccation adaptation hypothesis, assumes that costs of desiccation resistance are balanced by benefits in arid environments. Ectothermic vertebrates and insects living in arid environments tend to be more desiccation resistant and lose water more slowly than their mesic counterparts (Eckstrand and Richardson 1981; Gibbs and Matzkin 2001; Tracy et al. 2010). Even at smaller scales, such as within a habitat, tiger beetle species with higher desiccation resistance use drier microhabitats (Schultz and Hadley 1987). The tropical rainforest canopy and understory have distinct microclimates: The air temperature experienced by insects in the canopy averages 1 °C warmer than on the ground below; surface temperatures in the boundary layer – the air layer next to the surface – can average up to 10 °C warmer (Oke 1978; Kaspari et al. 2015). Here, we test the assumption that the vapor pressure deficit (VPD) – a measure of the drying power of the air – is higher in the canopy of a tropical forest, and contrast the desiccation resistance of canopy insects with litter insects.

Insects have a variety of mechanisms to reduce desiccation. First, larger insects tend to have lower surface area to volume ratio, more water storage, and more fat that can be converted to metabolic water (Hadley 1994). Within communities, larger species of fruit flies (Gibbs and Matzkin 2001), tiger beetles (Schultz and Hadley 1987), and ants (Hood and Tschinkel 1990) are more resistant to desiccation than their smaller congeners. Such examples largely come from the temperate zone. Here, we test the body size hypothesis in the tropics, where insects (e.g., *Drosophila* species) were found to have low desiccation resistance and low evolutionary potential for its increase (Hoffmann et al. 2003). Second, insects can also slow desiccation by simply having more water in their tissues (Hadley 1994). Canopy ants rely on more water‐based food, such as honeydew and extra‐floral nectar (Blüthgen et al. 2000), and are likely to have higher water content, and thus be pre‐adapted to living in environments with high VPDs. Third, insects can actively slow water loss by, for example, closing spiracles, or increasing rectal water reabsorption (Harrison et al. 2012). A simple test for such active regulation compares the water loss of dead and living individuals. We predict that active water loss regulation will be more prevalent in the tropical canopy compared to the cooler, moister understory.

An individual's desiccation resistance may also be constrained by other adaptations to the warm canopy such as thermal tolerance, measured as critical thermal maximum (CT_max_), the temperature at which animals lose the ability to control muscle contraction (Lutterschmidt and Hutchison 1997). We foresee two scenarios. First, if increased thermal tolerance and desiccation resistance require different costly investments, then this can result in trade‐off between one investment over the other, causing a negative correlation between desiccation resistance and CT_max_. For example, insects can prevent overheating through either passive (Lighton 1994) or active evaporative cooling (Heinrich 1980; Hadley et al. 1989). This allows them to tolerate higher temperatures but results in a high water loss (Renault et al. 2005). Second, as temperature and VPD are often positively correlated (Parker 1995), the same traits that favor desiccation resistance may also favor thermal tolerance. For example, increased body size may allow an insect to better survive both thermal and desiccation stress. If so, desiccation resistance and CT_max_ should be positively correlated.

Here, we use a dominant, diverse tropical insect assemblage to test a basic desiccation adaptation hypothesis and explore potential mechanisms for desiccation resistance.

## Materials and Methods

### Study site

We conducted our sampling in a lowland tropical wet forest, during the rainy season on Barro Colorado Island (BCI; 9°10′N, 79°51′W), Republic of Panama. Mean annual temperature is 27 °C, while mean annual rainfall is c. 2600 mm and largely occurs during the rainy season from May to December (Leigh 1999). So far, 350 ant species are recorded for this forest (Donoso personal communication). We identified ant species in the laboratory using an online database (evergreen.edu/ants/antsofcostarica.html), supplemented with a reference collection of BCI ants of the senior author. Voucher specimens are deposited in the laboratory of the senior author.

### Measuring microclimate

**Figure 1:**
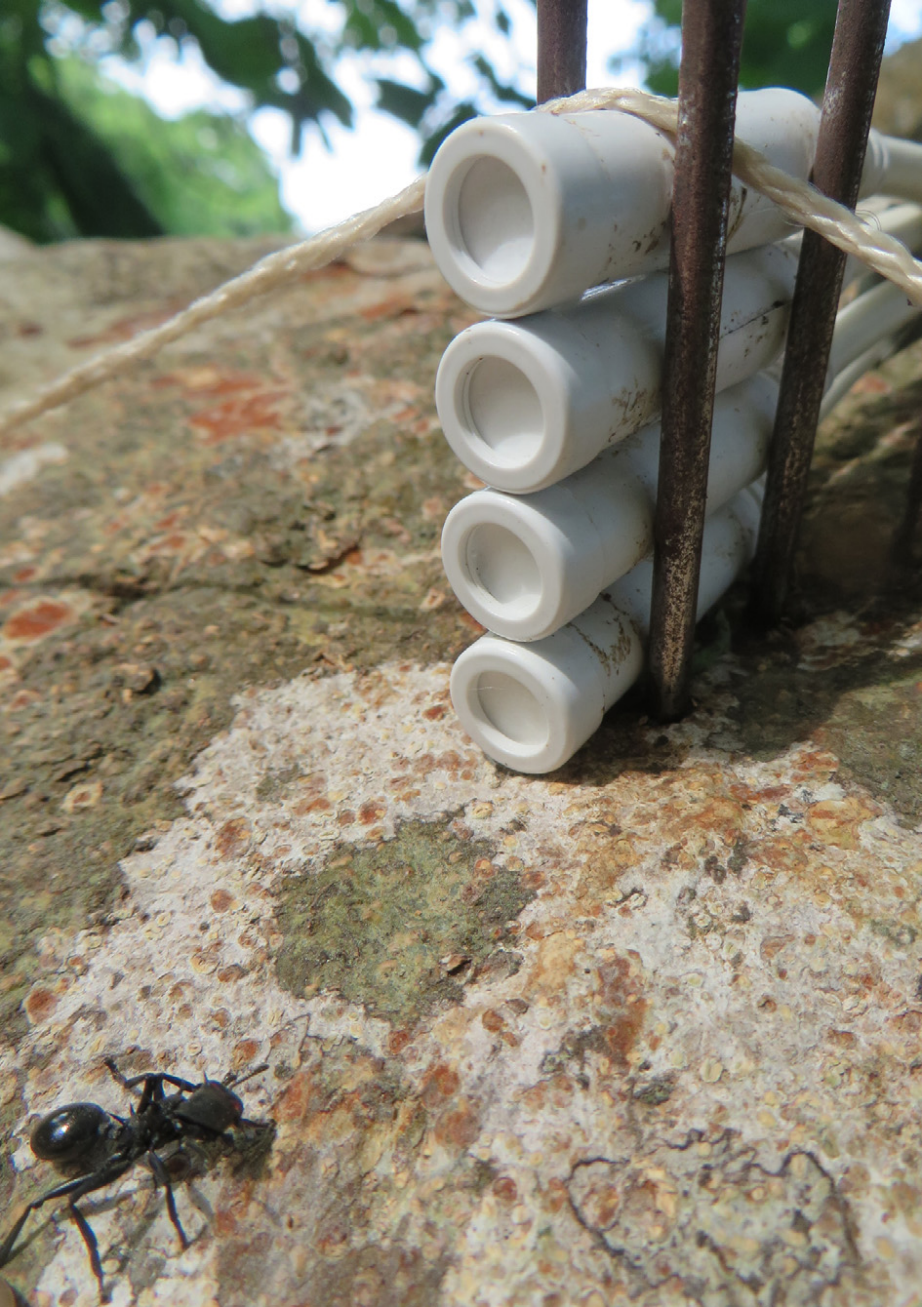
Worker of *Cephalotes atratus*, in a *Dipteryx panamensis* canopy next to the data loggers used for measuring temperature and relative humidity. *Cephalotes atratus* was the second largest canopy ant we tested.

We contrasted the temperature and VPD of canopy and understory microclimates of six tree species: *Anacardium excelsum, Bombacopsis quinata, Ceiba pentandra, Dipteryx panamensis, Jacaranda copaia,* and *Pseudobombax septenatum* that vary in their canopy architecture and their epiphyte load (Condit et al. 2010). We accessed the canopy using a single rope technique (Perry 1978). We placed HOBO Pro v2 (U23‐002) Temp/Relative Humidity Data Loggers in the canopy and the understory. We tied the base of the logger with a zip tie and attached the probe directly on a branch or a liana with polyester twine. Understory loggers were placed either in the leaf litter or on the understory vegetation. As the sensor was 10 mm in diameter, it estimated relative humidity and temperature at 0–10 mm above the surface, still exceeding the size of the large ants we tested (e.g., *Cephalotes atratus*, Fig. 1). We collected the data after 2 weeks of logging temperature and relative humidity in 10‐min intervals. We calculated actual VPD using our temperature and relative humidity measurements and formulae from Monteith and Unsworth (2007). We then calculated VPD as the difference between the saturation vapor pressure and actual vapor pressure, in kiloPascals (kPa). As VPD relies on both temperature and relative humidity, it is more biologically relevant than relative humidity alone (Anderson 1936).

### Measuring desiccation resistance

We measured desiccation resistance in 82 ant species from eight subfamilies: 34 from the canopy and 48 from the understory (Table S1). We collected the ants with an aspirator and tested them the same day. We considered all ants nesting and foraging in the canopy as canopy ants and ants nesting in the soil or litter as ground nesting ants regardless of their foraging preferences. Ants were collected from 1 to 10 colonies per species (median = 2), depending on species rarity. We placed five workers in glass Scintillation vials (1.5 cm in diameter) sealed with a mesh, next to which we attached a vial filled with fully dehydrated Drierite (W.A. Hammond Drierite Co. Ltd., Xenia, OH). We drilled a 1‐cm opening on the vial lids, which were then glued together with the mesh in between. Thus, the relative humidity in the ant vial was approximately 0%. We used 10‐mL glass vials for small ants and 20 mL for larger ants (time to death does not change with the vial volume: Kruskal–Wallis, *χ* ^2^ = 0.35, df = 1, *P* = 0.56). We monitored worker condition hourly, recording the time to death for each of the five workers. As a control, ant vials were connected to an empty vial (i.e., no Drierite). Species‐level desiccation resistance was measured as the average time of death of 2nd and 3rd workers (i.e., LT_50_).

### Measuring hydration and water loss of ants

We tested the prediction that canopy ants were more hydrated, and lost water more slowly than understory ants, using five large‐bodied common species from each habitat (larger species were easier to measure mass loss accurately). Foraging workers from the same colony were collected and weighed to 0.001 mg with a microbalance (Sartorius MC5), paired to be similar in weight, and one ant of the pair was killed by freezing at −80 °C. The pair was separately exposed to Drierite as above, the live ant checked every 30 min until it lost muscle control, and both ants were then weighed. Finally, both ants were dried in the oven at 60 °C and weighed to the nearest 0.001 mg to record their dry mass. Hydration of live ants is presented as a percent of water content at the outset of the experiment. Water loss is presented as the percent difference between wet mass at the outset and the end of desiccation trial for both live and dead ants.

### Measuring CT_max_

We measured the CT_max_ of each species with a digital dry bath (USA Scientific Thermal‐Lok 2‐position dry heat bath, advertised accuracy ±0.2 °C). We tested five workers from three different colonies for each species. Each worker was placed in an Eppendorf tube whose cap was filled with modeling clay and then loaded in the dry bath. Starting at 36 °C, we raised the temperature 2 °C every 10 min, until all workers lost muscle control. We used the temperature at which 50% of workers lost muscle contraction as our CT_max_. Ants used in these trials were oven dried at 60 °C and then weighed to the nearest 0.001 mg with a microbalance.

### Data analysis

All analyses were conducted using R version 3.2.2 (R Core Team 2015). We used two‐sample Wilcoxon tests to compare survival times of ants exposed to desiccant with the ants in control treatments, because data were non‐normal. The same test was used to compare the differences in temperature and VPD between the canopy and litter. We checked the data for normality using the Shapiro–Wilk W‐test. We used linear models to describe the relationship between the log_10_‐transformed LT_50_ and log_10_‐transformed body mass (using *lm* function in the R package *stats*). Linear models were also used when testing the relationship of ant LT_50_ in air and 0% humidity, when testing for the relationship of CT_max_ and body mass, and to analyze the relationship of LT_50_ and CT_max_. To test for the presence of outliers, we used Grubbs' test in the R package *outliers*.

We used generalized linear models (GLMs) to test the effect of body mass and habitat on ant desiccation resistance. We used an information‐theoretic approach to remove nonsignificant effects from the full model using probability values (see Zuur et al. 2013 for model selection details). The model comparison was based on ΔAIC values – the difference of the AIC of the *i*th model and the optimal model with lowest AIC value.

## Results

### How does vapor pressure deficit vary between canopy and litter?

Daily temperature 10 mm above the branch surface in the canopy of the focal tree species during day hours (6:00–18:00 h) averaged 1.1 °C higher than the temperature recorded in the understory (mean ± SD: 27.8° ± 2 °C vs. 26.7° ± 1.5 °C, Wilcoxon test *W* = 8.0 × 10^7^; *P* < 0.001). The difference reduced to 0.37 °C at night (18:00–6:00 h, *W* = 6.0 × 10^7^; *P* < 0.001). These differences were consistent across canopies of different tree species and their accompanying litter (Bujan unpublished data). Daily VPD from the same sensor averaged 0.38 kPa higher in the canopy (0.43 ± 0.37 kPa, *W* = 1.0 × 10^8^, *P* < 0.001; Fig. S1A) than in the understory (0.05 ± 0.11 kPa). This difference decreased to 0.15 kPa during the night when the canopy was on average wetter than during the day 0.16 ± 0.19 kPa, as was the understory (0.012 ± 0.05 kPa, *W* = 8.0 × 10^7^, *P* < 0.001, Fig. S1B).

### Testing the desiccation adaptation hypothesis

**Figure 2:**
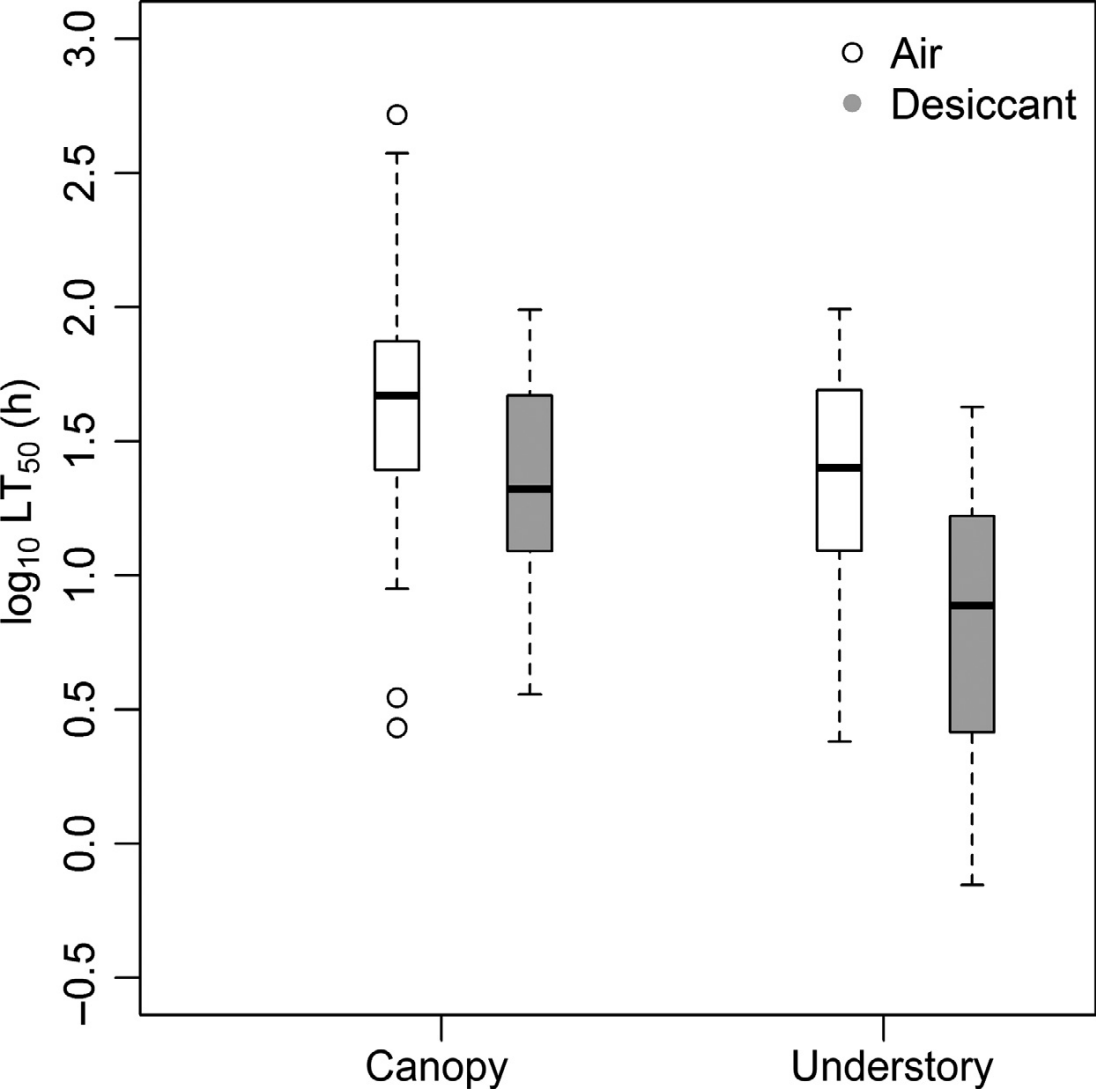
Log_10_ of lethal time (h) at which 50% of workers lost their muscle coordination (LT_50_), after they have been exposed to air (white) and desiccant (gray). The box and whisker plots are showing median of log_10_ LT_50_, upper and lower quartiles, as well as the maximum values and outliers.

We tested desiccation resistance of 82 ant species ranging from 0.01 to 52.70 mg in dry weight. Canopy ants from five subfamilies and 10 genera exposed to a desiccant survived almost three times longer than understory ants from seven subfamilies and 26 genera (LT_50_ = 32.2 ± 25.0 h vs. 11.5 ± 11, *W* = 1316, *P* < 0.001, Fig. 2). Canopy ants ranged from LT_50_ = 3.6 h (*Azteca chartifex* Emery, 1893) to 97.9 h [*Camponotus simillimus* (Smith, 1862)] while understory ants survived desiccation stress from LT_50 _= 0.7 h (*Trachymyrmex isthmicus* Santschi, 1931) to 42.5 h [*Pachycondyla harpax* (Fabricius, 1804)]. Control canopy ants survived 2.9 times longer in the air than when exposed to a desiccant (Fig. 2; *W* = 498, *P* = 0.01); understory ants survived twice as long (*W* = 205, *P* = 0.005). The increase between the difference of survival time in control and desiccation treatments increased with body mass (Fig. S2, LT_50control_ − LT_50dessicant_ = 0.39 mass + 1.19, *R*² = 0.24, *P* = 0.0005).

### Mechanism 1: Body size enhances desiccation resistance

**Figure 3:**
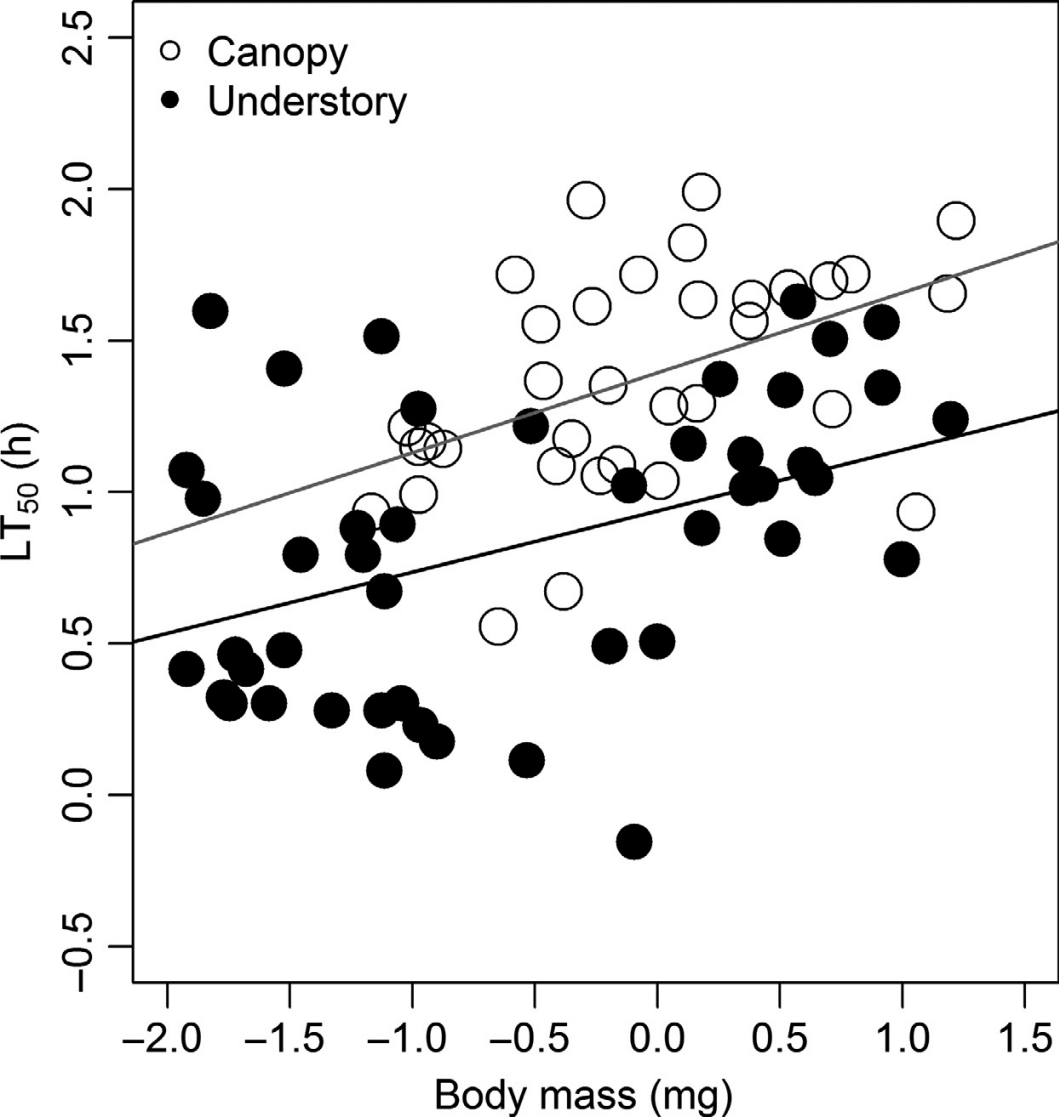
Relationship between species desiccation resistance (LT_50_) and log_10_ body mass (mg) in canopy and understory ants. Both linear models for this relationship differ significantly from a slope of 0 (see text for details): canopy – gray line, understory – black line.

Canopy ants were on average 16% heavier than litter ants (KW: *χ* ^2^ = 4.9, df = 1, *P* = 0.03). Desiccation resistance increased with body mass in both canopy and ground nesting ants (Fig. 3), and our linear model accounted for ca. ¼ of the variation (LT_50 _= 0.27mass + 1.2, *F*_1, 79 _= 24.8, *R* ^2^ = 0.24, *P* < 0.001). Body mass accounted for more variation in desiccation resistance of canopy ants (LT_50_ = 0.26mass + 1.4, *F*_1, 32 _= 9.1, *R* ^2^ = 0.22, *P* = 0.005), than for the understory ants (LT_50_ = 0.20 mass + 0.94, *F*_1, 45 _= 10.5, *R* ^2 ^= 0.19, *P* = 0.002). However, the slope and the variation in desiccation resistance explained by body mass in canopy and understory was not different from the values obtained at the community level. The optimal GLM model for explaining desiccation resistance includes both body mass and habitat as predictor variables and accounts for 42% of variation in desiccation resistance (Table S2).

There was a high variability in desiccation resistance within three genera with the most desiccation‐resistant species. Desiccation resistance of the genus *Camponotus* ranged from 10.9 to 97.9 h, in genus *Neoponera* species ranged from 17.4 to 78.8 h, and in *Cephalotes* species ranged from 18.8 h to 66.5 h (Table S1). Some litter genera, however, consistently had low desiccation resistance, for example *Pheidole* (1.5–4.7 h), and fungus‐growing ants, such as *Cyrphomyrmex* (1.7–2 h) and *Apterostigma* (Table S1, 1.3 h). When we used GLMs with genus as a predictor variable, in addition to habitat and mass, only the aforementioned fungus grower genera accounted for a portion of variation in desiccation resistance.

### Mechanism 2: Hydration enhances desiccation resistance

**Figure 4:**
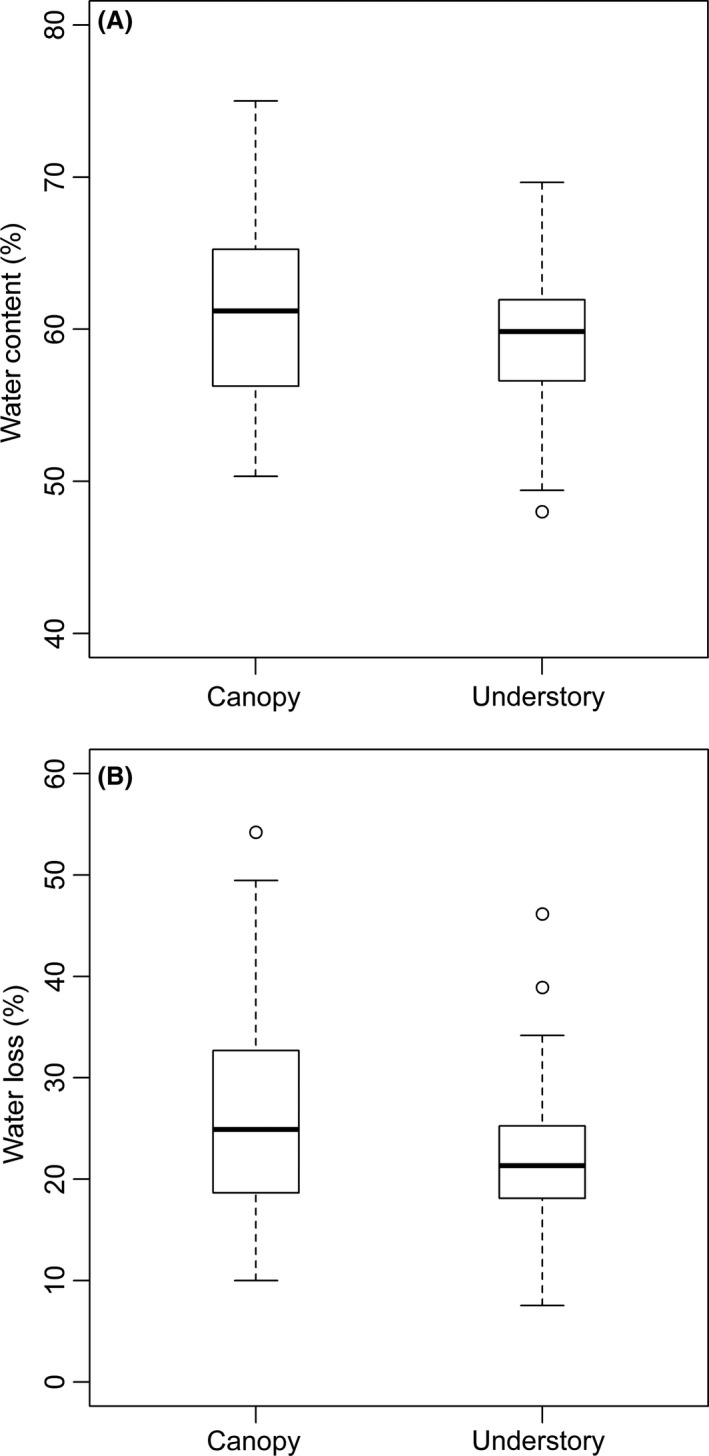
(A) Total water content (%) of canopy and ground nesting ants. (B) Total water loss (%) in canopy and ground nesting ants. The box and whisker plots are showing median of % water content (A) and % total water loss (B), upper and lower quartiles, as well as the maximum values and outliers.

We studied the role of hydration in desiccation resistance in 10 common ant species – five from each habitat – ranging in dry mass from 1.5 to 27.2 mg. These ants varied in % water content from 48% in *Eciton hamatum* (Fabricius, 1782) to 75% in *Camponotus sericeiventris* (Guérin‐Méneville, 1838), but % water was not related to body mass (*F*_1, 8 _= 0.004, *P* = 0.95). Water content of canopy ants averaged 2.5% higher than the water content of understory ants (Fig. 4A, 61.3 ± 6.0% vs. 58.8 ± 4.5%, *W* = 3280, *P* = 0.016). Water content, however, was not a good predictor of desiccation resistance (*F*_1, 8 _= 0.41, *P* = 0.54) or CT_max_ (*F*_1, 7 _= 0.017, *P* = 0.90).

Water loss was on average higher in the canopy than in the litter (Fig. 4B, 26.3 ± 10 vs. 22.1 ± 6.8, *W* = 3975, *P* = 0.01), but the average rate of ant water loss did not differ between the habitats (*W* = 4247, *P* = 0.54). Live and dead canopy ants did not differ in their total water loss under desiccation stress (*W* = 944, *P* = 0.17), nor did understory ants (*W* = 816, *P* = 0.56). Water loss rate of live canopy ants did not differ from their dead counterparts (*W* = 592, *P* = 0.70), same was true for the understory ants (*W* = 691, *P* = 0.90).

### Is there a trade‐off between desiccation resistance and thermal tolerance?

**Figure 5:**
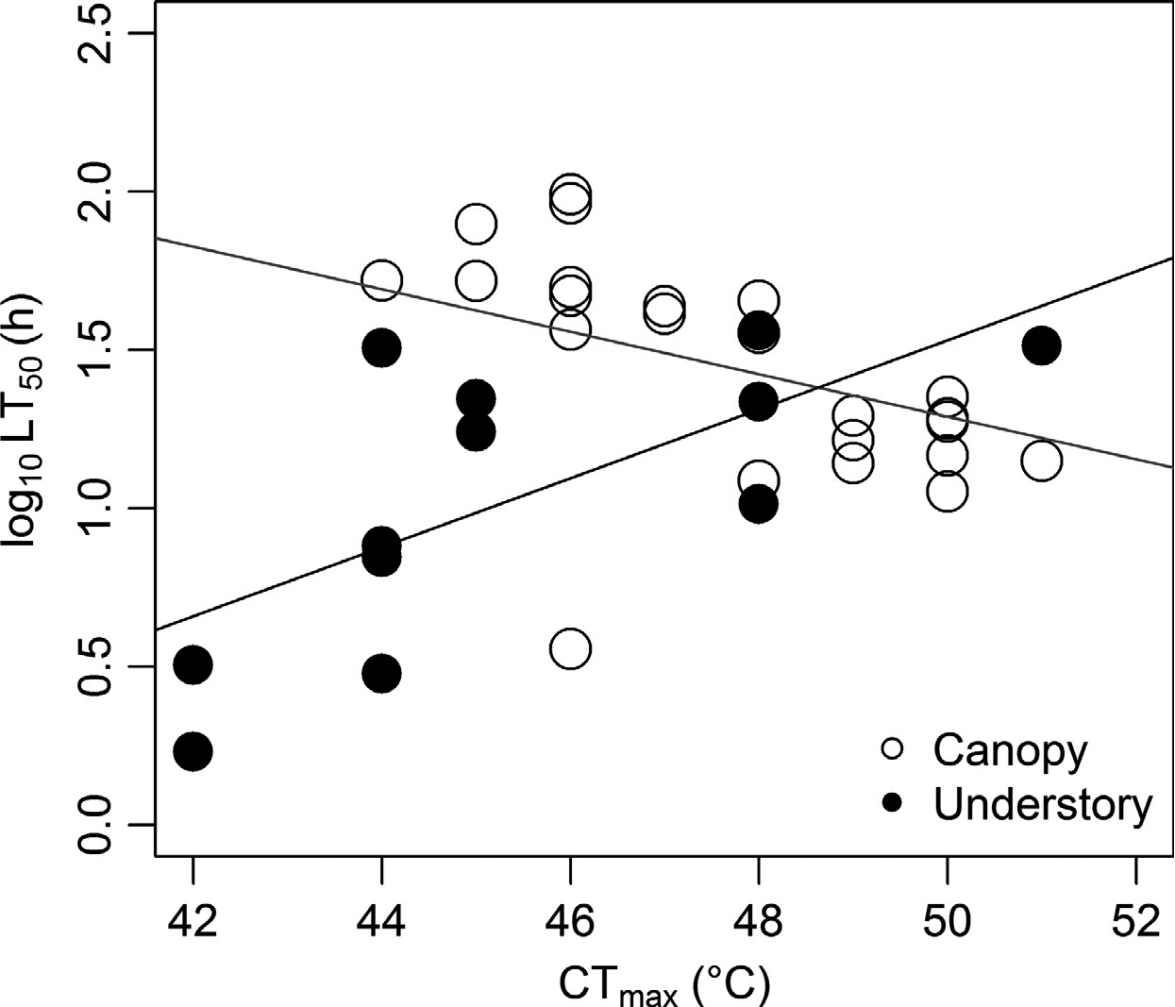
Relationship between desiccation resistance (LT_50_) and critical thermal maximum (CT_max_) in canopy and understory ants.

We did not find a consistent trade‐off between LT_50_ and CT_max_ (Fig. 5). After using body mass as a covariate in our linear models, CT_max_ and desiccation resistance correlated in opposite ways in canopy and litter ants. Desiccation resistance in canopy ants decreased with CT_max_ (*F*_2,20_ = 8.6, *R* ^2 ^= 0.46, *P* = 0.002). This relationship in canopy ants is even more pronounced when an outlier – *Azteca* cf. *chartifex*, was removed (*F*_2,19_ = 25, *P* < 0.001, *R* ^2 ^= 0.72). Understory ants, however, show a positive relationship between desiccation resistance and CT_max_ (*F*_2,9 _= 13, *R* ^2 ^= 0.74, *P* = 0.002).

## Discussion

Here, we show that desiccation can be a major challenge for small ectotherms even in a moist tropical forest. Daytime vapor pressure deficits were nine times higher in the canopy than in the understory below, and canopy ants tolerated desiccation stress three times longer than understory ants. Desiccation‐resistant ants tended to be larger, although less than predicted by changes in their surface area to volume ratio. Moreover, canopy ants tend to contain more water than the understory ants, suggesting a possible role of water storage in postponing desiccation. Combined with an earlier study on thermal tolerance (Kaspari et al. 2015), these data point to large differences in both the microclimate between canopy and understory and the resulting traits of a dominant insect group.

### Body size and desiccation resistance

Consistent with their lower surface area to volume ratios, larger insects typically have higher resistance to desiccation (Lighton et al. 1994; Chown and Nicolson 2004; Schilman et al. 2007; Harrison et al. 2012), but few studies include sufficient sample sizes to estimate the nature of this relationship. A notable exception is Hood and Tschinkel (1990), who found that across 25 ant species from a pine woodland and 11 ant desert species, desiccation resistance scaled to dry mass^0.55^, which differed significantly from the expected value of dry mass^0.67^. At the adjusted mass range, we examined 64 species and overall found an even weaker relationship with body mass (Fig. S3, *b *=* *0.33, *R* ^2 ^= 0.20). When we examine this relationship at the habitat level, both slopes were less steep than predicted (*b*_canopy _= 0.35 and *b*_litter_ = 0.32).

Differences in surface area to volume ratios did not sufficiently account for variation in desiccation resistance in this community. A number of factors may reduce this constraint. Ants might be using behavioral adaptations to avoid overheating and desiccation stress, as small ectotherms are more susceptible to microclimate variability, specifically temperature changes (Woods et al. 2015). For example, activity of smaller ants was higher at lower VPD, while larger ants showed no preference for VPD levels in a lowland rainforest in Costa Rica (Kaspari 1993). In the tropical canopy, epiphytes can provide a moister and cooler microclimate (Stuntz et al. 2002), which might allow canopy ants to behaviorally avoid desiccating. Finally, larger ants might be less desiccation resistant than predicted by the surface area to volume ratio because of potential trade‐offs between desiccation resistance and other traits like thermal performance (Baudier et al. 2015; Kaspari et al. 2015).

Overall, canopy ants of a subtropical pine woodland averaged eight times higher desiccation resistance than the understory ants (Hood and Tschinkel 1990); this difference was three times lower in our tropical forest. The larger difference between two habitats at higher latitudes arises because of a higher VPD in the pine forest canopy. This pattern of lower desiccation resistance of insects in the tropical regions has been thoroughly studied in *Drosophila* species, which have lower desiccation resistance in the tropics when compared to species from higher latitudes (Stanley and Parsons 1981; Karan et al. 1998; Hoffmann et al. 2003).

### Water content is not a good predictor of desiccation resistance

In xeric habitats, large ants contain more water and have higher desiccation resistance than smaller workers from the same colony (Lighton et al. 1994; Johnson 2000). We found no relationship between ant water content and body mass. Habitat was a better predictor of the total water content than body mass as canopy ants, relying on a more water‐based diet, averaged 2.5% higher than ground nesting ants. The total water content of both canopy (61%) and understory ants (59%) is similar to the water content measured for desert ant workers 66% *Pogonomyrmex rugosus* Emery, 1895 (Lighton and Feener 1989), and 63% *Pogonomyrmex occidentalis* (Cresson, 1865) (Johnson 2000). As water content was not a good predictor of CT_max_ or desiccation resistance, active evaporative cooling is likely not an efficient way of reducing body temperature in habitats with average relative humidity above 90%. Our results suggest that tropical ants do not use extra water to cope with desiccation or thermal stress. An absence of water content – body mass relationship in the ants we studied could be due to mass range we used. We note that by testing ants heavier than 1.5 mg, we excluded a large proportion of small ants.

### Ants did not differ in apparent ability to retain moisture

Contrary to our prediction, live canopy ants were not better at reducing water loss and had an overall 3.6% higher water loss rate than dead ants. Dead desert ants lose more water than live ants over time (Lighton et al. 1994), but this was not the case in any ants we tested. We found no significant differences in water loss between live and dead ants of either canopy or litter species (Fig. S4). Hood and Tschinkel (1990) also found no difference in water loss between live and dead ants in the higher latitude ant community. This suggests that canopy ants likely have other, passive, mechanisms for preventing water loss. For example, insects with less porous cuticles and those with more branched saturated lipids (Gibbs 2002) can reduce the cuticular respiration, which accounts for more than 80% of the water loss in insects (Quinlan and Gibbs 2006).

### Evidence for tradeoffs is complex

Our study shows the importance of examining the relationship between traits enabling survival in a set of coupled environmental conditions. Insect thermal sensitivity (Huey et al. 1991; Deutsch et al. 2008; Hurlbert et al. 2008; Diamond et al. 2012) and desiccation resistance are frequently studied independently (Hadley 1994; Chown 1993; Gibbs et al. 1997; Schilman et al. 2007) despite their potential to interact (Renault et al. 2005; Terblanche et al. 2006). Ectothermic vertebrates (Crowley 1987; Ladyman and Bradshaw 2003) and insects (Smith et al. 1999) often prefer lower temperatures under desiccation stress. We found an increase in desiccation resistance with CT_max_ in the understory, while ant species of the tropical canopy showed the opposite pattern: decreased desiccation resistance as their CT_max_ increases. One possible solution to this puzzle lies in the cuticular lipids that coat the exoskeleton and inhibit water loss (Hood and Tschinkel 1990). As temperature increases, these lipids eventually change their consistency and increase cuticular permeability (Gibbs 2002, 2011). Our findings suggest that in canopy ants, which experience some of the most extreme temperatures in the tropical forests (Kaspari et al. 2015), more permeable cuticle increases evaporative water loss. This in turn, allows ants in the hottest environments to engage in passive evaporative cooling and could be the reason why canopy ants with CT_max_ >46 °C have lower desiccation resistances. If true, the composition, quantity and physics of cuticular hyrocarbons, may prove a useful functional trait in predicting the thermal ecology and water balance ability of small invertebrates.

### Caveats

Our study quantifies the difference in microclimates during a tropical wet season. Dry season conditions of this tropical forest include higher VPD and higher temperatures than during the wet season (Leigh 1999). Ant activity in this forest is 25% lower during the dry season compared to the wet season (Kaspari and Weiser 2000). Furthermore, in drier conditions, desiccation resistance has been shown to increase in a fruit fly species (Hoffmann et al. 2005). Our study may thus underestimate desiccation resistance in this assemblage, and its seasonality.

We measured total water loss gravimetrically at the time of death for each ant species. Measuring water loss with a flow‐through or closed system respirometry would allow us to distinguish between excretory, respiratory, and cuticular water loss (Harrison et al. 2012). Continuous monitoring of water loss in live and dead ants would further allow us to test whether water loss regulation is present at the beginning of exposure to dry conditions in the canopy and litter ants.

### Future work

The desiccation adaptation hypothesis remains a powerful and relatively untested tool in global change biology and requires more validation across Earth's climates and invertebrate communities. Furthermore, the variety of mechanisms that can generate desiccation resistance, including fluidity of epicuticular lipids, deserve further study as a key functional trait in tiny ectotherms. Our works suggests that within any given ecosystem, a variety of microclimates exist, and within any given community, a diversity of mechanisms can interact to generate the distribution of desiccation resistance among individuals and between populations. Against this backdrop of interesting complexity, ecologists are called upon to predict responses to a likely world of increasing seasonal and multi‐annual drought in the subtropics and tropics (Fu 2015). One prediction arising from our work and that of Hood and Tschinkel (1990): The higher average desiccation resistance in canopy species suggests their increase at the expense of litter ants in a world of increasing droughts.

## Conflict of Interest

None declared.

## Supporting information


**Figure S1.** The difference in vapor pressure deficit (VPD) in the canopy and the litter during the day (A) and night (B).
**Figure S2.** Relationship between the difference of ant lethal time in the air and when exposed to the desiccant with respect to body mass. All the values were log_10_ transformed.
**Figure S3.** Relationship between desiccation resistance (LT_50_) and body mass on a log scale from our study (solid lines) compared to ants studied by Hood and Tschinkel (1990) – dashed lines.
**Figure S4.** Water loss (%) in different ant species examined.

**Appendix S1**
**Table S1.** List of ant species from two studied habitats used to measure workers' critical thermal maximum (CT_max_) and lethal time when exposed to the desiccant (LT_50_).
**Table S2.** Generalized linear models used to test the differences in desiccation resistance among 82 ant species.
