# Supplementary material for: Desiccation resistance in tropical insects: causes and mechanisms underlying variability in a Panama ant community
Source: Ecol Evol. 2016 Aug 8;6(17):6282–91. doi: 10.1002/ece3.2355 (PMC5016648; doi:10.1002/ece3.2355)
Supplement: Supplementary file 1 — Figure S1. The difference in vapor pressure deficit (VPD) in the canopy and the litter during the day (A) and night (B). Figure S2. Relationship between the difference of ant lethal time in the air and when exposed to the desiccant with respect to body mass. All the values were log10 transformed. Figure S3. Relationship between desiccation resistance (LT50) and body mass on a log scale from our study (solid lines) compared to ants studied by Hood and Tschinkel (1990) – dashed lines. Figure S4. Water loss (%) in different ant species examined. [file ECE3-6-6282-s001.docx]

# Supplemetal material online

Figure S1. The difference in vapor pressure deficit (VPD) in the canopy and the litter during the day (A) and night (B).

Figure S2. Relationship between the difference of ant survival in the air and when exposed to the desiccant with respect to body mass. All the values were log_10_ transformed.

Figure S3. Relationship between desiccation resistance (LT_50_) and body mass on a log scale from our study (solid lines) compared to ants studied by Hood and Tschinkel (1990) – dashed lines. Canopy – dashed gray: LT_50_ = 0.61mass + 0.89, R^2^ = 0.66, and understory – dashed black: LT_50_ = 0.45mass + 1.68, R^2^=0.63. At the adjusted mass range, in our community, canopy ants – gray solid: LT_50_ = 0.35mass + 1.43, R^2^ = 0.28, p = 0.002, and understory ants – black solid: LT_50_= 0.32mass + 0.92, R^2^ = 0.27, p = 0.001).

Figure S4. Water loss (%) in different ant species examined. Canopy species are shown in blue – light blue represents water loss in live ants, and dark blue in dead ants. Litter species are shown in gray: light – live ants, dark – dead ants. The box and whisker plots are showing median of % total water loss, upper and lower quartiles, as well as the maximum values and outliers. We tested the following species: *Pseudomyrmex gracilis* (PSEUgrac), *Dolichoderus bispinosus* (DOLIbisp), *Cephalotes atratus* (CEPHatra), *Cephalotes umbraculatus* (CEPHumbr), *Camponotus sericeiventris* (CAMPseri), *Eciton hamatum* (ECIThama), *Odontomachus bauri* (ODONbaur), *Ectatomma tuberculatum* (ECTAtube), *Ectatomma ruidum* (ECTAruid), *Atta colombica* (ATTAcolo).


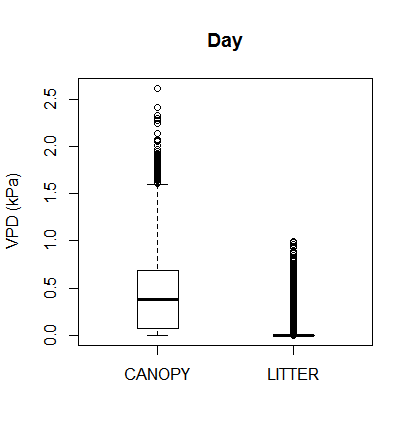
Figure S1A

Figure S1B


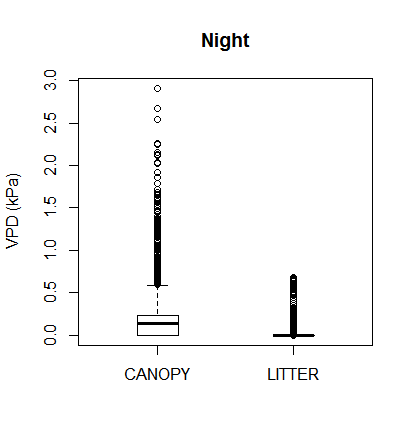


Figure S2


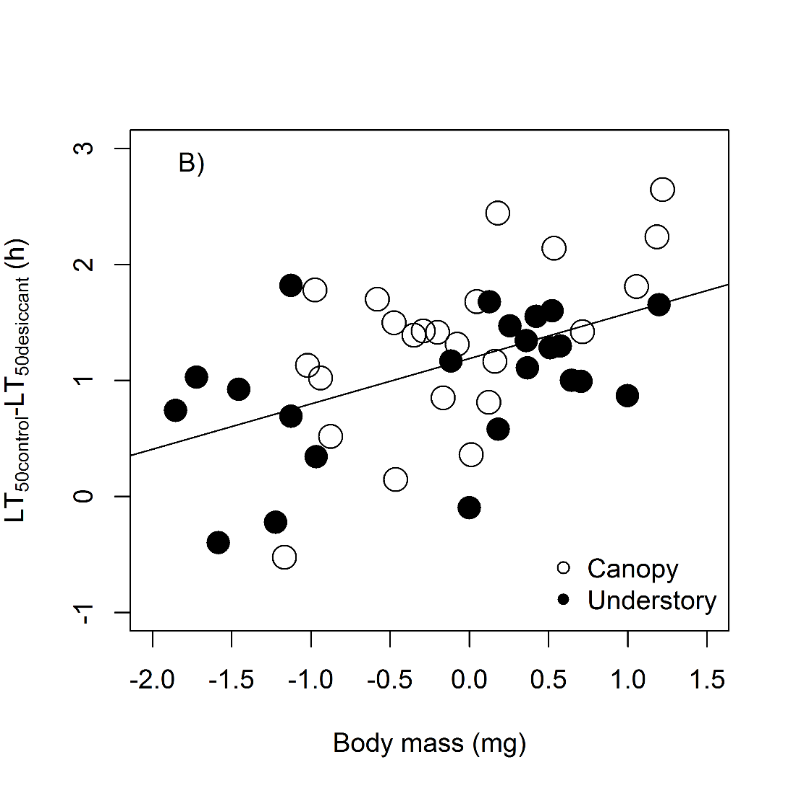


Figure S3


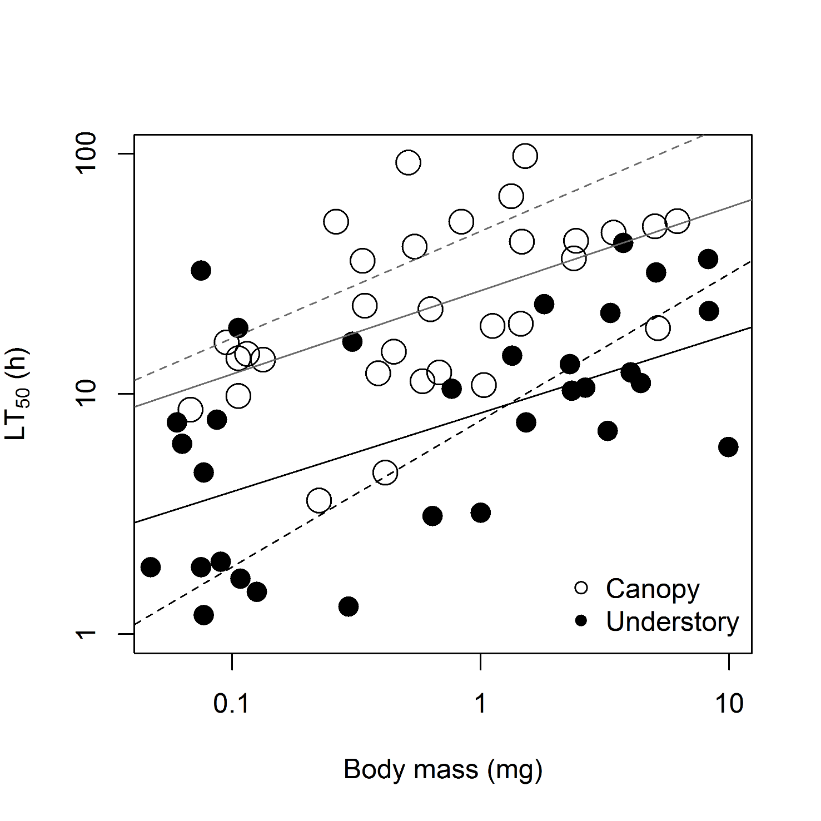


Figure S4
