# Supplementary material for: Desiccation resistance in tropical insects: causes and mechanisms underlying variability in a Panama ant community
Source: Ecol Evol. 2016 Aug 8;6(17):6282–91. doi: 10.1002/ece3.2355 (PMC5016648; doi:10.1002/ece3.2355)
Supplement: Supplementary file 2 — Appendix S1 Table S1. List of ant species from two studied habitats used to measure workers' critical thermal maximum (CTmax) and lethal time when exposed to the desiccant (LT50). Table S2. Generalized linear models used to test the differences in desiccation resistance among 82 ant species. [file ECE3-6-6282-s002.docx]

Appendix 1

Table S1. List of ant species from two studied habitats used to measure workers’ critical thermal maximum (CT_max_) and survival when exposed to the desiccant (LT_50_). The number of colonies (N_col_) refers to the number of colonies used to measure LT_50_.

| Subfamily | Genus | Species | HABITAT | LT_50_ (h) | N_col_ | CT_max_ |
| --- | --- | --- | --- | --- | --- | --- |
| Dolichoderinae | *Azteca* | *chartifex* | canopy | 3.6 | 3 | 46 |
| Dolichoderinae | *Azteca* | *_sp3_* | canopy | 12.2 | 10 | 48 |
| Dolichoderinae | *Azteca* | *pilosula* | canopy | 12.3 | 1 | - |
| Dolichoderinae | *Dolichoderus* | *bispinosus* | canopy | 19.6 | 4 | 49 |
| Dolichoderinae | *Dolichoderus* | *Debilis* | canopy | 22.5 | 5 | 50 |
| Dolichoderinae | *Dolichoderus* | *laminatus* | canopy | 43.1 | 1 | - |
| Formicinae | *Brachymyrmex* | *longicornis* | canopy | 8.6 | 1 | - |
| Formicinae | *Camponotus* | *_JTL056_* | canopy | 10.9 | 1 | - |
| Formicinae | *Camponotus* | *_JTL044_* | canopy | 35.8 | 2 | - |
| Formicinae | *Camponotus* | *novogranadensis* | canopy | 41.1 | 4 | - |
| Formicinae | *Camponotus* | *sericeiventris* | canopy | 49.8 | 1 | - |
| Formicinae | *Camponotus* | *sanctaefidei* | canopy | 52.1 | 1 | - |
| Formicinae | *Camponotus* | *linnaei* | canopy | 52.2 | 1 | 45 |
| Formicinae | *Camponotus* | *brevis* | canopy | 91.9 | 3 | 46 |
| Formicinae | *Camponotus* | *simillimus* | canopy | 97.9 | 4 | 46 |
| Myrmicinae | *Acromyrmex* | *volcanus* | canopy | 8.6 | 2 | - |
| Myrmicinae | *Cephalotes* | *umbraculatus* | canopy | 18.8 | 2 | 50 |
| Myrmicinae | *Cephalotes* | *atratus* | canopy | 45.2 | 4 | 48 |
| Myrmicinae | *Cephalotes* | *minutus* | canopy | 66.5 | 2 | - |
| Myrmicinae | *Crematogaster* | *stollii* | canopy | 4.7 | 1 | - |
| Myrmicinae | *Crematogaster* | *tenuicula* | canopy | 13.9 | 6 | 49 |
| Myrmicinae | *Crematogaster* | *carinata* | canopy | 14.1 | 10 | 51 |
| Myrmicinae | *Crematogaster* | *brasiliensis* | canopy | 14.7 | 1 | 50 |
| Myrmicinae | *Crematogaster* | *limata* | canopy | 16.4 | 4 | 49 |
| Myrmicinae | *Procryptocerus* | *belti* | canopy | 11.3 | 2 | 50 |
| Ponerinae | *Neoponera* | *carinulata* | canopy | 36.7 | 2 | 46 |
| Ponerinae | *Neoponera* | *unidentata* | canopy | 43.4 | 5 | 47 |
| Ponerinae | *Neoponera* | *striatinodis* | canopy | 46.9 | 9 | 46 |
| Ponerinae | *Neoponera* | *_jtl13_* | canopy | 52.4 | 1 | 44 |
| Ponerinae | *Neoponera* | *bugabensis* | canopy | 78.8 | 5 | 45 |
| Pseudomyrmecinae | *Pseudomyrmex* | *_spPSW01_* | canopy | 9.8 | 1 | - |
| Pseudomyrmecinae | *Pseudomyrmex* | *viduus* | canopy | 15 | 2 | - |
| Pseudomyrmecinae | *Pseudomyrmex* | *boopis* | canopy | 19.2 | 1 | 50 |
| Pseudomyrmecinae | *Pseudomyrmex* | *oki* | canopy | 23.3 | 6 | - |
| Dolichoderinae | *Tapinoma* | *melanocephalum* | litter | 6.2 | 3 | - |
| Dolichoderinae | *Technomyrmex* | *fulvus* | litter | 18.8 | 1 | - |
| Ecitoninae | *Eciton* | *hamatum* | litter | 7 | 2 | 44 |
| Ecitoninae | *Eciton* | *dulcium* | litter | 10.6 | 2 | - |
| Ecitoninae | *Eciton* | *burchellii* | litter | 11.1 | 1 | - |
| Ecitoninae | *Labidus* | *praedator* | litter | 3.2 | 1 | 42 |
| Ecitoninae | *Nomamyrmex* | *esenbeckii* | litter | 6 | 2 | - |
| Ectatomminae | *Ectatomma* | *ruidum* | litter | 21.7 | 3 | 48 |
| Ectatomminae | *Ectatomma* | *tuberculatum* | litter | 36.4 | 2 | 48 |
| Ectatomminae | *Gnamptogenys* | *regularis* | litter | 14.4 | 1 | - |
| Formicinae | *Brachymyrmex* | *coactus* | litter | 16.7 | 1 | - |
| Formicinae | *Brachymyrmex* | *heeri* | litter | 39.6 | 1 | - |
| Formicinae | *Camponotus* | *_JTL004_* | litter | 22.1 | 1 | 45 |
| Formicinae | *Paratrechina* | *guatemalensis* | litter | 1.2 | 1 | - |
| Formicinae | *Paratrechina* | *longicornis* | litter | 7.8 | 3 | - |
| Myrmicinae | *Apterostigma* | *_JTL015_* | litter | 1.3 | 1 | - |
| Myrmicinae | *Atta* | *cephalotes* | litter | 7.6 | 2 | - |
| Myrmicinae | *Atta* | *colombica* | litter | 10.3 | 1 | 48 |
| Myrmicinae | *Crematogaster* | *flavosensitiva* | litter | 32.6 | 2 | 51 |
| Myrmicinae | *Cyphomyrmex* | *rimosus* | litter | 1.7 | 7 | 42 |
| Myrmicinae | *Cyphomyrmex* | *costatus* | litter | 1.9 | 1 | - |
| Myrmicinae | *Cyphomyrmex* | *major* | litter | 2 | 2 | - |
| Myrmicinae | *Pheidole* | *_lash9_* | litter | 1.5 | 1 | - |
| Myrmicinae | *Pheidole* | *_sp2_* | litter | 1.9 | 1 | - |
| Myrmicinae | *Pheidole* | *multispina* | litter | 2 | 2 | - |
| Myrmicinae | *Pheidole* | *_sp1_* | litter | 2.1 | 1 | - |
| Myrmicinae | *Pheidole* | *mendicula* | litter | 2.6 | 1 | - |
| Myrmicinae | *Pheidole* | *harrisonfordi* | litter | 2.9 | 4 | - |
| Myrmicinae | *Pheidole* | *rugiceps* | litter | 3 | 3 | 44 |
| Myrmicinae | *Pheidole* | *bicornis* | litter | 4.7 | 1 | - |
| Myrmicinae | *Sericomyrmex* | *amabilis* | litter | 3.1 | 1 | - |
| Myrmicinae | *Solenopsis* | *_lash5_* | litter | 2.6 | 1 | - |
| Myrmicinae | *Solenopsis* | *_JTL002_* | litter | 9.5 | 2 | - |
| Myrmicinae | *Solenopsis* | *terricola* | litter | 11.8 | 7 | - |
| Myrmicinae | *Strumigenys* | *_sp2_* | litter | 2 | 1 | - |
| Myrmicinae | *Strumigenys* | *_sp1_* | litter | 6.2 | 1 | - |
| Myrmicinae | *Strumigenys* | *gundlachi* | litter | 25.5 | 1 | - |
| Myrmicinae | *Tetramorium* | *bicarinatum* | litter | 16.5 | 1 | - |
| Myrmicinae | *Trachymyrmex* | *isthmicus* | litter | 0.7 | 1 | - |
| Myrmicinae | *Wasmannia* | *auropunctata* | litter | 7.6 | 6 | 44 |
| Paraponerinae | *Paraponera* | *clavata* | litter | 14.5 | 2 | - |
| Ponerinae | *Hypoponera* | *_JTL002_* | litter | 10.5 | 1 | - |
| Ponerinae | *Leptogenys* | *_JTL007_* | litter | 12.3 | 1 | - |
| Ponerinae | *Leptogenys* | *_jelSUL_* | litter | 13.3 | 1 | - |
| Ponerinae | *Leptogenys* | *punctaticeps* | litter | 23.6 | 2 | - |
| Ponerinae | *Odontomachus* | *bauri* | litter | 32 | 4 | 44 |
| Ponerinae | *Neoponera* | *villosa* | litter | 17.4 | 1 | 45 |
| Ponerinae | *Pachycondyla* | *harpax* | litter | 42.5 | 1 | - |

Table S2. Generalized linear models used to test the differences in desiccation resistance among 82 ant species. Habitat and body mass were used as predictor variables. For each model degrees of freedom, AIC values, ΔAIC and AIC weights are listed. Model terms include all the terms present in the model in question.

| MODEL TERMS | Df | AIC | ΔAIC | AIC Weights |
| --- | --- | --- | --- | --- |
| Mass + Habitat | 4 | 82.3 | 0 | 1 |
| Mass | 3 | 102.2 | 19.9 | 0 |
| Habitat | 3 | 98.9 | 16.6 | 0 |
| Null | 2 | 122.9 | 40.6 | 0 |
|  |  |  |  |  |
|  |  |  |  |  |
|  |  |  |  |  |
|  |  |  |  |  |
